# Practical randomly selected question exam design to address replicated and sequential questions in online examinations

**DOI:** 10.1007/s40979-022-00103-2

**Published:** 2022-04-12

**Authors:** Ahmed M. Elkhatat

**Affiliations:** Department of Chemical Engineering, Qatar University, PO Box 2713, Doha, Qatar

**Keywords:** Exam integrity, Question pool exam, Randomly selected questions exam

## Abstract

Examinations form part of the assessment processes that constitute the basis for benchmarking individual educational progress, and must consequently fulfill credibility, reliability, and transparency standards in order to promote learning outcomes and ensure academic integrity. A randomly selected question examination (RSQE) is considered to be an effective solution to mitigate sharing of questions between students by addressing replicated inter-examination questions that compromise examination integrity and sequential intra- examination questions that compromise examination comprehensivity. In this study, a Monte Carlo approach was used to design six examination schemes for the purpose of generating and evaluating 600 RSQEs in order to investigate the effects of RSQE design on replicated inter-examination and sequential and intra-examination questions. Results revealed that the number of randomly selected questions from the pool and the number of sub-pools inversely affected the replication and sequencing of the examination questions. Thus, by designing the RSQE in many sub-pools, in equivalence to the number of examination questions and selecting only one question from each sub-pool, and updating the sub-pools after each examination, the passing of information can be prevented, ensuring the integrity of the examinations.

## Introduction

Exams are part of the assessment processes that benchmark individuals’ educational progress and should be conducted in a way that promotes learning outcomes and upholds academic integrity. Ensuring academic integrity within online examinations has become a chief concern for educators. One such way of safeguarding academic integrity is by adopting methods to mitigate rampant breaches of the online examination procedures (Balasubramanian, DeSantis, & Gulotta, 2020; Dendir & Maxwell, 2020; Fask, Englander, & Wang, 2014), that were primarily developed due to the COVID-19 pandemic confinement (Clark et al., 2020; Dicks, Morra, & Quinlan, 2020; Jacobs, 2021). An underdeveloped sense of academic integrity and lax/absence of deterrence enforced by the educational institution preparing the examination can be a principal reason for cheating among students (Lang, 2014). Online examinations misconduct is accessible due to lack of faculty observation and prevalence of the internet – facilitating fact (i.e., answer) searching, especially if the actual examination questions were already available and gathered from online sources (Burgason, Sefiha, & Briggs, 2019; Kennedy, Nowak, Raghuraman, Thomas, & Davis, 2000).

In order to mitigate examination misconduct and question-sharing, a few educators suggested using proctoring technologies, such as webcams and microphones, to track and record students during the examination. Despite the effectiveness of such proctoring technologies in alleviating academic dishonesty during online examinations, they have limitations that are considered demanding in terms of not only cost and technical requirements, but also in terms of social and psychological implications on students (Karim, Kaminsky, & Behrend, 2014; Kharbat & Abu Daabes, 2021; Nigam, Pasricha, Singh, & Churi, 2021). Thus, to circumvent these drawbacks of procuring technologies, educators indicated the designing of examination questions to mitigate cheating and answer-sharing. Suggestions involved developing examination questions using open-ended questions or take-home examinations as effective solutions (Bengtsson, 2019; Schmidt-McCormack, Fish, Falke, Lantz, & Cole, 2019). These questions involve higher levels of student-thought and analysis, resulting in differing answers, enabling the instructor to analyze text-matching (similarity indexing) to safeguard academic integrity. Nevertheless, concerns are associated with examiner bias, thus offering a legal argument by a non-passing-graded student. Moreover, students can compromise the integrity of written essays (Bengtsson, 2019; A. M. Elkhatat, K. Elsaid, & S. Almeer, 2021b; Schuwirth & Van Der Vleuten, 2004).

Other suggestions include the development of examination questions ‘from scratch’, or paraphrasing a question that could prevent searching for questions (and related answers) online (A. Elkhatat, K. Elsaid, & S. Almeer, 2021a; Golden & Kohlbeck, 2020). Although this approach appears practical, students can breach the examination procedures by sharing the questions and answers with their classmates, searching on tutoring websites (e.g., Chegg) (Lancaster & Cotarlan, 2021; Steel, 2017), or hiring on-demand independent experts (e.g., tutors) to help students online. However, students mostly resort to sharing examination questions rather than tutoring services as tutoring services can have a different approach to solutions than what is taught in the class, which the instructor can consider an indication for academic misconduct (A. Elkhatat et al., 2021a). In contrast, classmates mostly use the same solution style taught. Furthermore, due to a typographical issue, tutoring websites may direct pupils to wrong responses (Donovan, 2020).

Hence, educators suggest using a test of randomly selected questions from a vast question bank (pool) as an effective solution to address question-sharing (A. Elkhatat et al., 2021a; Imran et al., 2019; Ware, Kattan, Siddiqui, & Mohammed, 2014). In a randomly- selected-questions examination (RSQE), the educator creates a question pool containing similar-value questions and specifies the number of questions from that pool to be given in the examination. In RSQE, every student gets a differing selection of questions - even if the examination allows multiple attempts, each attempt will probably contain a novel selection of questions.

Currently, all online-learning management systems allow for the creation of RSQEs. These learning management systems use differing names for the random selection feature, where RSQE is termed in Blackboard® (Blackboard, n.d.), USA as a ‘Random Block,’ and in Canvas®, USA, it is described as a ‘Question Group’. RSQE has plenty of advantages; it can be applied to any type of question, such as multiple-choice questions (MCQs), essay questions, true or false questions, among others. Educators can assign the correct answer for various questions (e.g., ordering, filling in the blank, matching, multiple answers, multiple-choice, Likert, true/false, etc.) using the online learning management system. As a result, without the intervention of the examiner, the examinations are evaluated automatically. Essay and file answer questions, on the other hand, need the examiner’s judgment and grading.

It is worth mentioning that RSQE interferes with students’ collective memory, which allows them to recall a recently finished test from memory and share the questions with other students who have not taken the exam yet (Persky & Fuller, 2021). Although RSQE allows randomly selected questions, online-learning management systems do not track the selected questions since the question-selection process follows mathematical probability concepts. Hence, a proportion of all questions in the question pool might appear to many students, while other questions do not appear at all. This inter-examination repetition of questions allows for question-sharing between students undertaking the same examination. Another major concern is that the RSQE might allow for the selection of sequential questions from the question pool. Consequently, sequential questions can lead to an unfair/skewed distribution of questions within the online examination paper (OEP). Accordingly, RSQE should be designed effectively in order to eliminate/minimize replicated inter-examination questions as well as sequential intra-examination questions.

### Literature review

Although the definition of academic integrity is complex and primarily based on consensus, most universities define it as a commitment to several fundamental values, including honesty, trust, fairness, respect, and responsibility in learning, teaching, and research (“International Center for Academic Integrity. Fundamental Values Project.,” 2014; “Universities Australia. Academic Integrity Best Practice Principles,” 2017). Breaching of academic integrity includes breaches of the examination procedures (UniSA, 2022). Online examination misconduct can occur in a spectrum of manners, though the most predominant cheating practices are searching for the examination questions/question-related answers online together with question/answer-sharing between students.

Examination misconduct not only results in graduates with a shallow understanding of the subject knowledge, though such individuals are also more likely to engage in dishonorable behaviors to succeed throughout their future careers (Hodgkinson, Curtis, MacAlister, & Farrell, 2015). Multiple reasons encourage a student to breach integrity in the online examinations, including the shortage of understanding of the topic, lack of interest in studying, failure to manage the required examination time, immature feeling of academic integrity, and lack of rigorous deterrence against academic misconduct. (Lang, 2014). The rampant dishonesty incidents during online examinations have triggered educators and researchers to investigate cheating behavior and develop novel methodologies to prevent (or at least minimize) such educational loopholes to ensure academic integrity and assessment quality within online examinations.

It is noteworthy that fostering self-transcendent ideals through the existence of honor codes might minimize contract cheating (McCabe & Trevino, 1993); however, self-transcendence fails with ingroup loyalty. While students consider online searching for examination answers as cheating, their mindset is that question/answer-sharing constitutes ‘healthy collaboration’ and ‘ingroup loyalty’ among students (Jang, Lasry, Miller, & Mazur, 2017; Pulfrey, Durussel, & Butera, 2018). Due to the development of strong friendships, students experience a sense of ‘group loyalty to their peers’ (Wentzel, Barry, & Caldwell, 2004). Ingroup loyalty causes students to excuse collective cheating by claiming that “sharing is caring” (Pulfrey et al., 2018) and “good teamwork” (Jang et al., 2017) makes cheaters feel less ethically detached. Pulfrey and colleagues (Pulfrey et al., 2018) conducted an insightful study with 615 undergraduate university students to investigate how societal and individual competition affects collective cheating, respectively and how the degree of acquaintance with classmates affects collective cheating to understand the essential incentive of collective cheating better and share questions with classmates. The results showed that collective cheating fell dramatically by showing pupils a macro social competition image, albeit at the price of individual cheating. The individual competition also showed disengagement towards collective cheating at the expense of individual cheating. In addition, collective cheating increased among students who knew each other more than students of strangers. Another study explored students’ perceptions of cheating and its popularity (Honz, Kiewra, & Yang, 2010). The most prevalent and relevant findings of this study are that students consider sharing and giving information less of an ethical deviation than receiving information, and cheating outside campus is regarded by these students as less harsh of an ethical breach than cheating on campus.

Numerous studies suggested and developed different methodologies to mitigate such educational misconduct. The employment of proctoring technologies, such as webcams and lockdown browsers, to control cheating is one of the solutions that has been evaluated for such purposes (Karim et al., 2014; Kharbat & Abu Daabes, 2021; Nigam et al., 2021). The proctoring technologies can also include lockdown browsers that restrict the student’s computer, preventing the student from copying, pasting, or using other browsers until the end of the examination, or – alternatively – implement JavaScripts that can identify participant switching to additional browser/s. However, proctoring technologies obstruct students while taking the examination. Case in point, using lockdown browsers prevents students from using any other software on their computer terminal that might be required to answer the specific examination question at hand. Another concern relating to browser lockdown is that the examination-taker can cheat through the employment of a separate device, unless the examination is not proctored by camera/microphone surveillance. Karim and colleagues (Karim et al., 2014) conducted an exploratory study on 582 randomly-assigned participants for a remote technology-proctored examination. The results of this study implied that, although the approach effectively decreased cheating, it could unintentionally affect student reaction due to increased anxiety and privacy concerns. Another recent, systematic review on proctoring systems (Nigam et al., 2021), focused on artificial-intelligence-based and non-artificial intelligence-based proctoring systems, together with the essential parameters for their design. The study raises several ethical concerns related to proctoring technology, including the risk of reducing fairness levels – typically associated with artificial intelligence judgment - in addition to the attenuation of student privacy and autonomy. In agreement with these studies, Kharbat and Abu Daabes (Kharbat & Abu Daabes, 2021) analyzed 815 attempts within 21 online examinations to evaluate how well students performed under technology-proctored examinations. Their research findings highlighted the negative environmental and psychological factors that impact students, including feelings of stress and anxiety during the examination time-frame and students’ significant concern regarding privacy invasion. In essence, despite the effectiveness of such proctoring technologies in mitigating cheating during online examinations, previous literature reveals concerns on anxiety and privacy during the examination time-frame. Furthermore, limitations of the proctoring techologies in terms of cost and technical requirements, add additional challenges for proper implementation of such technologies.

Several studies have scrutinized written-assignment examinations as another approach to address cheating in such circumstances. Written-assignment examinations include open-ended questions or take-home examinations. Bengtsson (Bengtsson, 2019) conducted a systematic review on take-home examinations in higher education. The study concluded that take-home examinations are only recommended for higher-order Bloom’s taxonomy levels that involve higher-order thinking skills - including analysis, synthesis, and evaluation. Nevertheless, academic integrity might be breached by a proportion of students. Consequently, take-home examinations should be avoided for lowest-order Bloom’s taxonomy levels that involve knowledge and comprehension. The review addressed the advantages and disadvantages of take-home examinations, their risks, and how such risks could be mitigated. The benefits of take-home examinations consisted in reducing student anxiety and promoting the learning experience through assessment, which fostered the educational process beyond memorization. Notwithstanding, the majority of reviewed research articles agree that take-home examinations can be easily compromised by unethical student behavior, including the engaging of a third-party proxy to perform the examination instead. Elkhatat and colleagues (A. M. Elkhatat et al., 2021b) provided scenarios of student-employed methodologies for plagiarizing their written assignments without becoming flagged by similarity indexing software packages. This study analyzed the effectiveness of nine academic-level similarity indexing products against these unethical breaching of academic integrity through the plagiarism of previous literature.

In contrast to previous approaches to mitigate cheating and question-sharing, few articles discussed RSQEs. The merit of RSQE is that it applies to any educational level - primary, secondary, or tertiary, and for any study subject, such as mathematics, science, history, among others. Moreover, it helps instructors design both lower-order and higher-order thinking questions according to Bloom’s taxonomy (Bloom, 1956). Lower-order thinking questions include remembering information, demonstrating understanding, and using acquired information, while higher-order thinking questions include analyzing, discovering, and organizing information, integrating knowledge, and making judgments. Online learning management systems allow educators to design and develop essential and guiding questions to measure higher-order thinking (Blackboard). Ali (Ali, 2011) suggested randomly-selected questions with vast question pools as a strategy to counter cheating through question-sharing. However, as a method for mitigating question-sharing and student memorizing the bank questions, the researcher proposed a hybrid model of 30% randomly-selected questions and 70% non-randomly selected questions.

Notably, a vastly expanded question pool is not synonymous with a reduction in replicated inter-examination questions, since the frequency of one random event from multiple events could be higher than expected due to the probability – as described by the ambiguous issue recognized as the ‘Birthday Paradox’ (Swadling, 2019). Similarly, the frequency of sharing an identical question from a larger pool of questions can be higher than expected, leading to question repetition among students undertaking a specific, identical examination. The probability of sharing question-sharing can be calculated according to the following equation;

$$ P=1-\frac{N!}{\left(N-x\right)!\ast {N}^x} $$

Where, N is the pool size, x is the number of selected questions from the pool.

Based on probability calculations, Wentworth’s Institute of technology’s teaching and learning perspectives forum (Cookel, 2015) provides precious guidelines on designing RSQEs to minimize the number of replicated inter-examination questions. The study calculated the probability of five questions selected from differing question pool sizes (10, 25, 50, 100, and 200 questions). The study introduced the concept of the ‘Birthday Paradox’ to predict the likelihood of no repeated questions from question pools. Nevertheless, this study did not provide information on the frequency of replicated inter-examination questions, which is essential when considering methods to mitigate question-sharing among students. Moreover, such probability calculations assume that the selection of questions is a fair event, which might not be correct and consequently requires an experimental study to prove it. In addition, it does not provide statistical information on the issue of sequential questions.

Moreover, no studies have investigated the sequential questions that can lead to an unfair/skewed distribution of the exam questions. Case in point, if an examiner designed the examination to consist of 10 randomly-selected questions from a pool of 100 questions, there is the distinct probability of two (or more) sequential questions to be selected from the same question pool. Having sequential questions from a question pool might be a concern when the examiner follows a patterned order when creating the specific question pool. One scenario is when the first number of questions are derived from one specific lecture/lesson (e.g., lecture #1), followed by another set of questions from the next lesson (e.g., lecture #2), with this pattern building the entire online examination paper (OEP).

Consequently, although active research is currently underway within the field of online examination design, no previous literature has yet focused on the effectiveness of differing RSQE designs to address the issues of replicated inter-examination questions or sequential intra-examination questions. This study aimed to fill this research vacuum using the Monte Carlo approach (James, 1980), by conducting an empirical study through the development of 600 RSQEs - to investigate the impact of RSQE design in resolving such educational challenges.

## Methodology

An empirical study using Monte Carlo analytical approach was implemented to investigate the impact of RSQE design on replicated inter-examination and sequential intra-examination questions.

The empirical study was performed in three main steps: (1) six RSQE proposals were designed; (2) 100 RSQEs were generated for each proposal, and results were recorded (total *n* = 600); (3) results and data analyses.

### **Step 1:** examination design

As previously described, all online-learning management systems allow the generation of RSQEs under different names, and such RSQEs can be applied to any question-type, educational level, and subject. This study employed the ‘Random Block’ (Blackboard®) to generate the RSQE. Initially, 100 questions (*n* = 100, coded from Q1 to Q100) were created using the platform’s ‘test tool’. This coding helped in tracking the appearance-list of questions within each generated examination. Although the platform’ test tool’ allows the generation of any question format, this study chose true/false-type questions. Since this study aimed to track replicated / sequential questions, consequently, the question format does not dictate the random-selection process. In addition, the true/false question format was the least complex and most rapid option for this study. Following the generation of the 100 coded questions, sub-pools and six RSQE designs were created, as indicated in Table 1. These six designs aimed to investigate (1) the ratio of selected questions / question pool on the replicated inter-examination and sequential intra-examination questions; (2) the number of sub-pools on the replicated inter-examination and sequential intra-examination questions.

**Table 1:** RSQE Designs

QPR	Exam Design	No of sub-pools	No of questions in each pool	No of the selected questions in each pool	Sub-pool questions
10%	Design 1	10	10	1	Sub-pool 1: Q1-Q10 Sub-pool 2: Q11-Q20 Sub-pool 3: Q21-Q30 Sub-pool 4: Q31-Q40 Sub-pool 5: Q41-Q50 Sub-pool 6: Q51-Q60 Sub-pool 7: Q61-Q70 Sub-pool 8: Q71-Q80 Sub-pool 9: Q81-Q90 Sub-pool 10: Q91-Q100
	Design 2	5	20	2	Sub-pool 1: Q1-Q20 Sub-pool 2: Q21-Q40 Sub-pool 3: Q41-Q60 Sub-pool 4: Q61-Q80 Sub-pool 5: Q81-Q100
	Design 3	2	50	5	Sub-pool 1: Q1-Q50 Sub-pool 2: Q51-Q100
	Design 4	1	100	10	Q1-Q100
5%	Design 1	5	20	1	Sub-pool 1: Q1-Q20 Sub-pool 2: Q21-Q40 Sub-pool 3: Q41-Q60 Sub-pool 4: Q61-Q80 Sub-pool 5: Q81-Q100
	Design 2	1	100	5	Q1-Q100

Two Questions/Pool Ratio (QPR) were considered in designing the RSQE; 10% QPR - in which the examination consisted of 10 randomly selected questions from a pool of 100 questions - and 5% QPR, in which the examination consisted of five randomly selected questions from a pool of 100 questions. The reason for considering only two QPRs in this study was that a higher percentage would increase the probability of question repetition, and any lower percentage would be a demanding task for an instructor to create a vastly expanded question pool.

As shown in Table 1, the first exam design of 10%QPR consists of ten sub-pools; each sub-pool contains ten questions. The ‘Random Block’ of Blackboard randomly picks one question from each pool to generate a ten-question exam. The other exam designs of the same 10%QPR consist of fewer sub-pool; the second design consists of five sub-pools, each containing 20 questions, and used to generate a ten-question exam by randomly picking two questions from each sub-pool. The third design of 10%QPR consists of two sub-pools containing 50 questions, while the fourth design contains only one sub-pool of 100 questions, from which the ten questions were randomly chosen. A similar approach was used to generate exams of 5%QPR, in which the first design consists of two sub-pools; each sub-pool contains 20 questions. The ‘Random Block’ of Blackboard randomly picks one question from each pool to generate a five-question exam, while the second design contains only one sub-pool of 100 questions, from which the five questions were randomly chosen.

### **Step 2:** generation of examinations and result recording

**Fig. 1:**
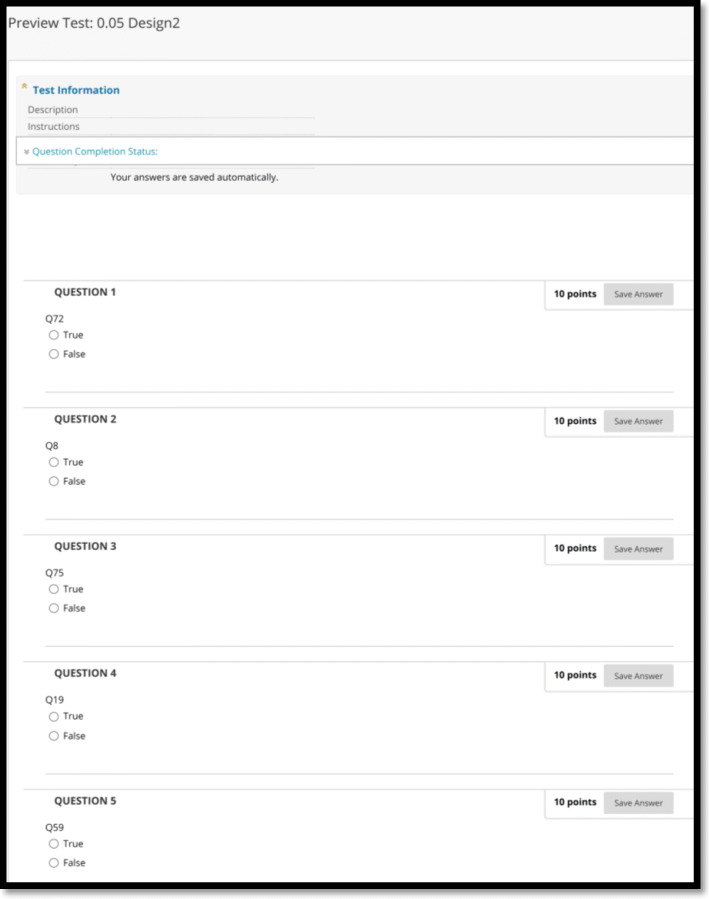
Example of generated exam

**Fig. 2:**
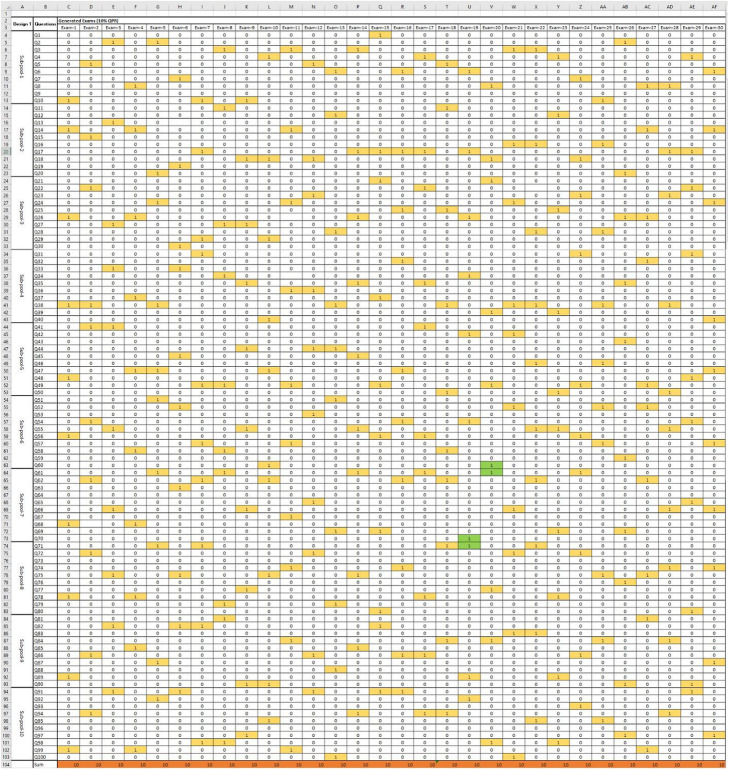
Recording of questions using Excel

The six RSQE examination designs were activated on the Blackboard™ platform, and each examination has had 100 attempts. An example of the generated examination is shown in Fig. 1. Each generated examination was analyzed using Microsoft Excel® 2016 [Microsoft™,USA], shown in Fig. 2. Questions from 1 to 100 were allocated to the first column (B), while the generated 100 examinations for each RSQE were assigned to columns (C-CX) in the worksheet, with individual worksheets dedicated to a single RSQE design. Numbers (0 or 1) were used to record the appearance-list of questions within each generated examination to facilitate the recording of the replicated and sequential questions, and to eliminate any potential errors during the recording process.

The percentages of replicated inter-examination questions (from the 100 generated examinations for each design) were determined. In addition, the percentages of sequential intra-examination questions were also determined. Sequential questions were categorized into:

- sequential duplicate questions (SDQ), in which two sequential questions (e.g., Q1 and Q2) coincided together in the same examination,
- sequential triplicate questions (STQ), in which three sequential questions (e.g., Q1, Q2, and Q3) coincided together in the same examination
- sequential quadratic questions (SQQ), in which four sequential questions (e.g., Q1, Q2, Q3, and Q4) coincided together in the same examination.

### **Step 3:** results data analyses

The ‘descriptive statistics package’ and the ‘histogram package’ of Microsoft Excel® 2016 were used to evaluate the statistics of all 600 examinations. The statistics included mean, median, mode, standard error, standard deviation, sample variance, range, minimum, maximum, kurtosis, skewness, and histogram of frequency.

Minimum and maximum values were the lowest-and highest-observed repeated questions, respectively, and can discern if the repetition in questions has a comparable value or else vary significantly. Statistical criteria’ mean,’ ‘median,’ and ‘mode.’ are used to determine the distribution skewness. When mean,’ ‘median,’ and ‘mode’ are equals, the distribution is symmetric. However, when ‘mean’ and ‘median’ are greater than ‘mode,’ the distribution is positively skewed, indicating a flatter right side. Conversely, when ‘mean’ and ‘median’ are less than ‘mode,’ the distribution is negatively skewed, showing a flatter left side. The positive or negative skewing of data distribution can be demonstrated by ‘box and whisker analysis’, which is also helpful in indicating whether there are unusual observations (outliers) in the data set. The difference between ‘box and whisker analysis’ and normal distribution is that in ‘box and whisker analysis,’ the data are distributed in a box in which ‘median value locates in the box (i.e., 50%) of the data, while box’s right and left edges represents the second (lower 25%) and third quartiles (upper 75%) of the data included. The Left and right whiskers represent the lower and upper outliers.

Another helpful statistical indicator is ‘kurtosis,’ which measures the impact of extreme observations / outliers on data distribution. Kurtosis indicates whether the data points scatter in peak or tails. If data points scatter in peak rather than tails, the distribution is (positive kurtosis) or (Leptokurtic) - characterized by heavy tails. However, (negative kurtosis) or (Platykurtic) is characterized by a flat peak - with dispersed data points having lighter tails (Joanes & Gill, 1998). It is noteworthy that, in the current study, the greater (positive skewness) and (negative kurtosis) were favorable for RSQE design as it indicated a higher frequency of the low-replicated questions and non-significant outliers.

## Results and discussion

This study aimed to answer two research questions relating to RSQE design, namely:

(1) How does RSQE design impact replicated inter-examination questions?

(2) How does RSQE design impact sequential intra-examination questions?

In order to answer each question, two points were considered:

1. the ratio of selected questions/question pool on the replicated inter-examination and sequential intra-examination questions (QPR)
2. the number of sub-pools on the replicated inter-examination and sequential intra-examination questions.

In order to facilitate data analysis and discussion, the six RSQE designs were categorized into two groups, namely the 10% QPR group (that included four designs, according to the number of sub-pools used to build the examination) and the 5% QPR group (that included two designs, according to the number of sub-pools), as shown in Table 1**.**

The 600 examination trials that were generated, underwent statistical analyses according to the above two categories. Table 2 demonstrates the statistics for replicated questions of the six RSQE designs, while Table 3 shows the statistics of sequential questions of the six RSQE designs.

**Table 2:** Descriptive statistics of replicated questions of RSQE Designs

QPR	Descriptive statistic	Mean	Standard Error	Median	Mode	Standard Deviation	Sample Variance	Kurtosis	Skewness	Range	Minimum	Maximum
10%	Design 1	10.00%	0.33%	10.00%	9.00%	3.31%	0.11%	−0.23	0.23	16.00%	2.00%	18.00%
	Design 2	10.00%	0.34%	10.00%	9.00%	3.37%	0.11%	0	0.21	17.00%	3.00%	20.00%
	Design 3	10.00%	0.31%	10.00%	8.00%	3.14%	0.10%	−0.31	0.24	16.00%	3.00%	19.00%
	Design 4	10.00%	0.30%	10.00%	11.00%	2.98%	0.09%	−0.39	0.16	14.00%	4.00%	18.00%
5%	Design 1	5.00%	0.23%	5.00%	4.00%	2.29%	0.05%	−0.59	0.26	10.00%	1.00%	11.00%
	Design 2	5.00%	0.22%	5.00%	4.00%	2.19%	0.05%	0.21	0.53	11.00%	1.00%	12.00%

**Table 3:** Statistics of sequential questions of RSQE Designs

QPR		Sequential Question	Max	Average	Min
10%	Design 1	SDQ	20.00%	1.00%	0.00%
		STQ	0.00%	0.00%	0.00%
		SQQ	0.00%	0.00%	0.00%
	Design 2	SDQ	60.00%	8.80%	0.00%
		STQ	30.00%	0.30%	0.00%
		SQQ	0.00%	0.00%	0.00%
	Design 3	SDQ	40.00%	13.20%	0.00%
		STQ	30.00%	0.30%	0.00%
		SQQ	0.00%	0.00%	0.00%
	Design 4	SDQ	60.00%	12.20%	0.00%
		STQ	30.00%	0.90%	0.00%
		SQQ	40.00%	0.40%	0.00%
5%	Design 1	SDQ	0.00%	0.00%	0.00%
		STQ	0.00%	0.00%	0.00%
		SQQ	0.00%	0.00%	0.00%
	Design 2	SDQ	40.00%	8.00%	0.00%
		STQ	60.00%	0.60%	0.00%
		SQQ	0.00%	0.00%	0.00%

As shown in Table 2**,** the minimum percentage of repeated questions increased proportionally with decreasing of sub-pools and QPP%. Similarly, sample variance and standard deviation that discern the percentage distribution of repeated questions increased proportionally with increasing sub-pools in both QPP% designs. Further to this, kurtosis showed negative values that decrease with increasing of sub-pools in 10%QPR.

On the other hand, Table 3 interprets the statistical features (minimum, average, and maximum) of sequential questions, namely SDQ, STQ, and SQQ. The percentage of sequential questions declined significantly with increasing sub-pools and decreasing QPP%.

### Research question 1 analysis

**Fig. 3:**
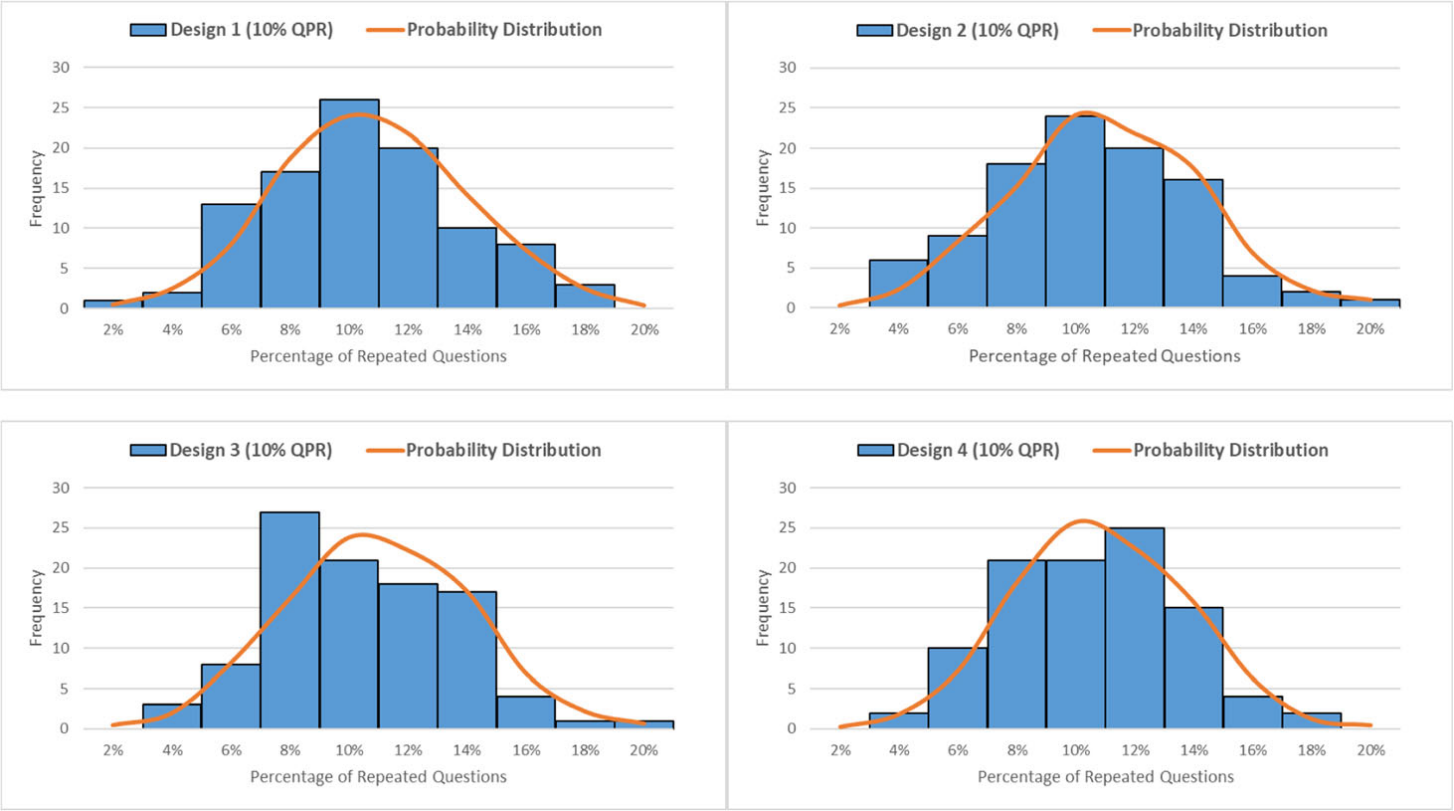
Histogram and Probability Distribution of 10%QPR in the four exam designs

**Fig. 4:**
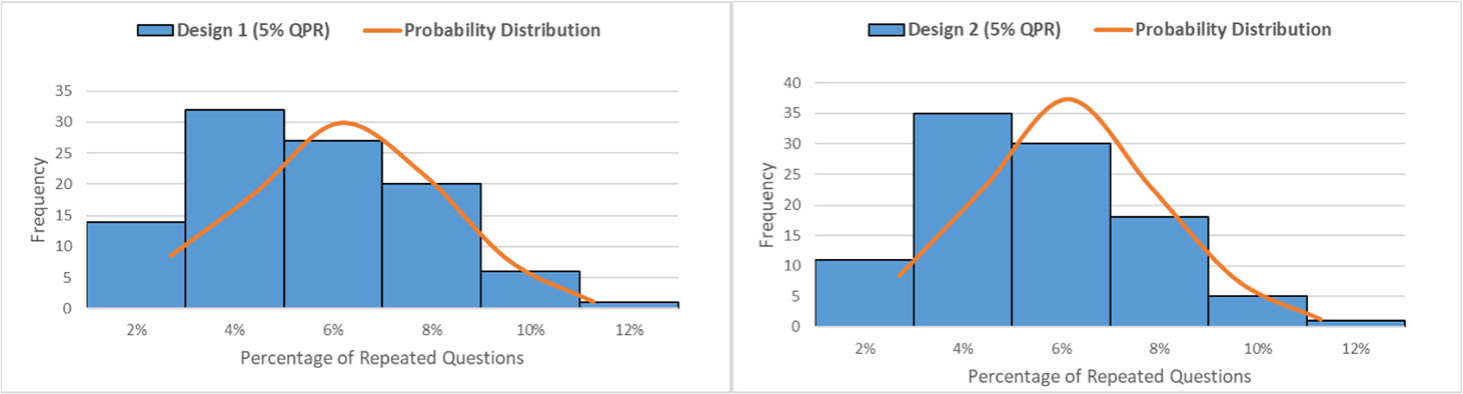
Histogram and Probability Distribution of 5%QPR in the two exam designs

The analysis of replicated inter-examination questions assesses how many times each question in the pool was repeated when the design was generated 100 x fold. The higher-replicated questions reflected an increased probability of question-sharing between classmates. The descriptive statistics of the 100 questions (Q1-Q100) in the four designs of 10%QPR and the two designs of 5%QPR are indicated in Table 2, and their repetition histogram and probability distribution are shown in Figs. 3 and 4.

Within the 10%QPR category, the standard deviation for 10% QPR/Design 4 (of one sub-pool) was 3.14%, and this increased proportionally with increasing of sub-pools, to 3.31% in the 10% QPR/Design 1 (of ten sub-pools). This increase in standard deviation from 2.91% to 3.31% reflects the tendency of asymmetrical frequency of replicated questions, by increasing the number of sub-pools within the RSQE. Skewness and kurtosis analyses indicated that this asymmetrical frequency of replicated questions trended towards the frequency with few-replicated questions. The increase of skewness positivity in design 1 compared with designs 2, 3, and 4 indicated that the tendency of few-replicated questions was higher than that of highly-replicated questions.

**Fig. 5:**
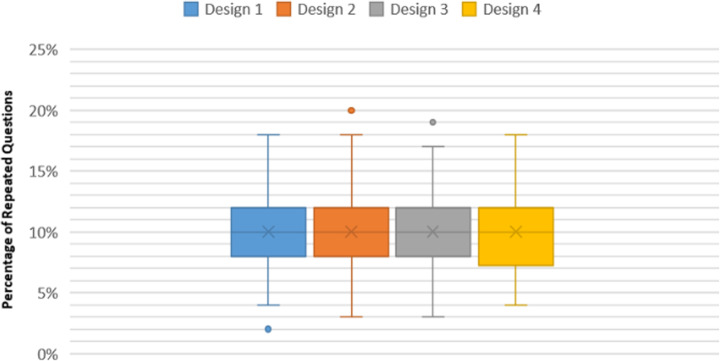
Box and whisker analysis of 10%QPR in the four exam designs

The negative kurtosis in the four RSQE designs of 10% QPR indicates a flat peak with non-significant outliers (Platykurtic distribution). However, the negative value of kurtosis analysis decreased, from 10% QPR/Design 4 to 10% QPR/Design, revealing a decrease in minor outliers. The positive skewness in data distribution was confirmed by box-and-whisker analysis, as shown in Fig. 5.

Notably, although the number of sub-pools in 10%QPR did not affect the average frequency of replicated questions, it positively affected having increased numbers of questions with fewer replications, which helped to address the issue of question-sharing between classmates.

**Fig. 6:**
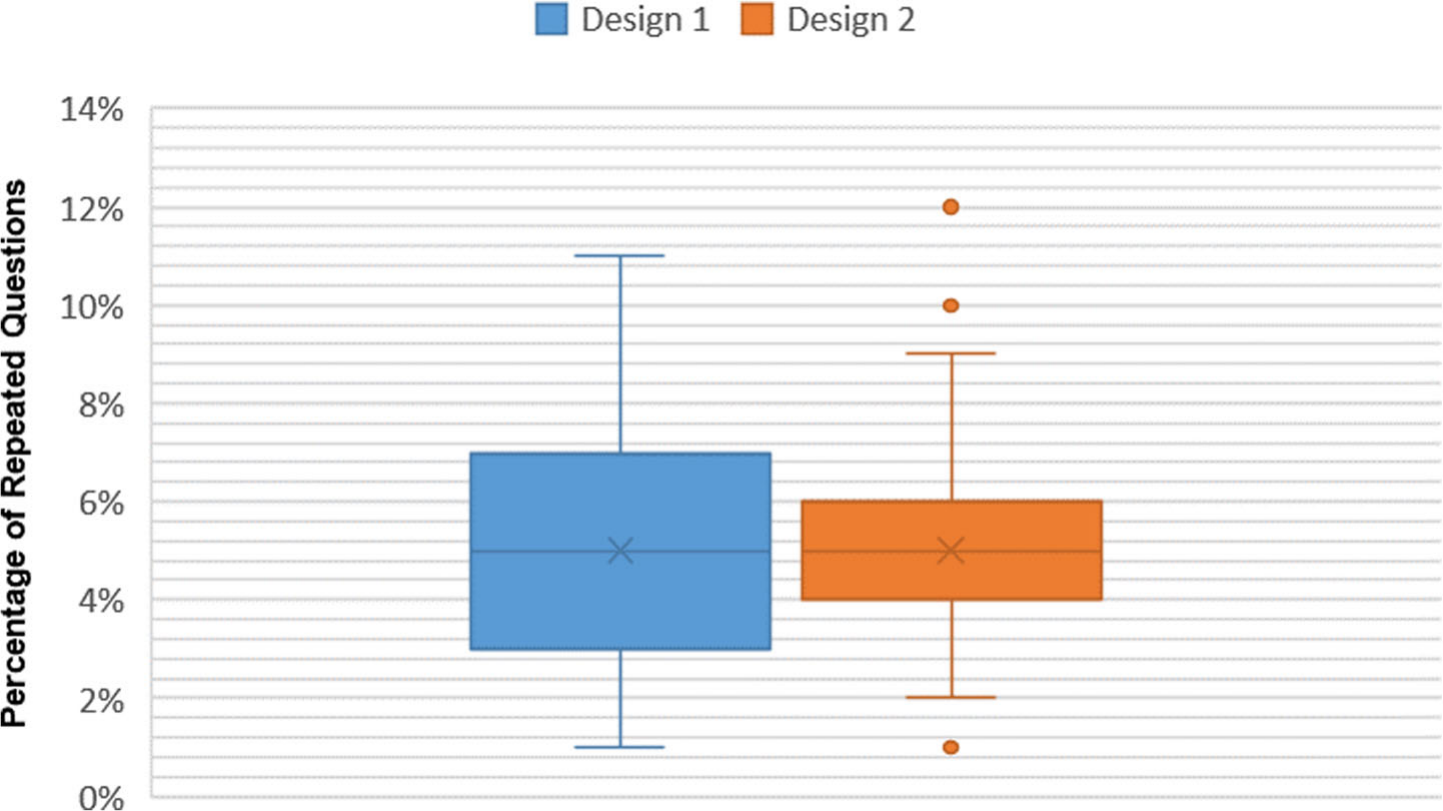
Box and whisker analysis of 5%QPR in the two exam designs

Conversely, in the 5%QPR category, the average frequency of replicated questions was inferior to the 10%QPR category. The standard deviation of 5% QPR/Design 2 (of one sub-pool) was 2.91%, and this increased with increasing the number of sub-pools, to 2.29% in 5% QPR/Design 2 (of five sub-poos), reflecting the tendency of asymmetrical frequency of replicated questions, by increasing the number of sub-pools in the RSQE. Skewness and kurtosis analyses indicated that this asymmetrical frequency of replicated questions trended toward the frequency with few-replicated questions. The two designs were positively skewed, reflecting a higher number of questions with low replicates. A positive ‘kurtosis’ was revealed in 5% QPR/Design 2, reflecting a leptokurtic distribution, characterized by significant outliers. The histogram and probability distribution showed skewness in the data (Fig. 4**)**, and was confirmed by box-and-whisker analysis in Fig. 6***.***

Consequently, it can be concluded that the number of questions that are randomly selected from the pool affects their replication at inter-examination level: where lower QPRs lead to less-replicated questions. Moreover, as the number of sub-pools increases, the tendency of increased questions with fewer replications is amplified.

### Research question 2 analysis

The percentage of SDQ, STQ, and SQQ in 10% and 5% QPRs categories are indicated in Table 3. In the 10%QPR category, designs 2, 3, and 4 demonstrated a high percentage of sequential questions. SDQ reached 60% of the examination questions in designs 2 and 4, and 40% in design 3. In addition, STQ reached 30% in designs 2,3 and 4, though was absent in design 1. SQQ only appeared once in design 4 and was absent in the other designs. Consequently, design1 effectively mitigated the sequential questions, as the design did not exhibit any STQ or SQQ questions, with mninimal levels of SDQ (mean of 1%). Similarly, in 5% QPR examinations (Table 3**)**, design 2 exhibited few occurrences of SDQ and STD, with an average of 8% and 0.3%, respectively. In contrast, design 1 did not show any sequential questions.

Hence, it can be concluded that the number of questions that were randomly selected from the pool affected their sequential questions in each examination; a reduction in QPR leads to reduced sequential questions. Moreover, as the number of sub-pools increased, the tendency of sequential questions was reduced.

## Discussion and tips for effective RSQE design

Ensuring academic integrity within online examinations has become a chief concern for educators who adopt different methods to address the rampant cheating in online examinations. Despite limitations, many of these methods (such as proctoring technologies and fostering self-transcendent ideals through applying honor codes) effectively reduce individual cheating. Notwithstanding, they fail with collective cheating, in which students share exam questions and answers with their classmates or use their collective memory to recall exam questions and pass them to other students who have not yet taken the exam. Due to the development of solid friendships, students experience a sense of ‘group loyalty to their peers, which cause students to excuse collective cheating by claiming that “sharing is caring” and that sharing and giving information is less of an ethical deviation than receiving information, making them feel less ethically detached.

Hence, randomly- selected-questions examination (RSQE) can be considered as an effective solution to address question-sharing and interfere with students’ collective memory to recall exam questions. In RSQE, every student gets a differing selection of questions from the question pool - even if the examination allows multiple attempts, each attempt will probably contain a novel selection of questions. However, a large question pool is not synonymous with a reduction in replicated inter-examination questions due to the ‘Birthday Paradox’. Furthermore, online learning management systems do not track the selected questions from a pool, resulting in a proportion of all questions in the question pool might appear to many students. In contrast, other questions do not appear at all. In addition to replicated inter-examination questions, RSQE may result in sequential intra- examination questions that compromise examination comprehensivity.

Therefore, this study aimed to design a practical RSQE to mitigate replicated inter-examination and sequential intra- examination questions by applying the Monte Carlo approach on produced 600 RSQE using six examination designs. This study revealed that the number of randomly selected questions from the question pool affected their replication at the inter-examination level: reduced QPR led to reductions in replicated questions. Moreover, as the number of sub-pools increased, the trend of additional questions with fewer replications increased. Furthermore, RSQE design impacted the sequential intra-examination questions - reduced QPR led to fewer sequential questions, and as the number of sub-pools increased, the trend of sequential questions decreased.

Reflecting on these results, the instructor can design RSQE to measure both lower-order and higher-order thinking skills. The following strategy is helpful for a proper RSQE design;

1. The instructor is advised to follow the following RSQE design: Create sub-pools equal to the number of exam questions, in which each sub-pool has questions representing 10% of students (For example, if the class has 50 students, each sub-pool should contain five questions). Then, pick one question from each sub-pool.
2. The instructor is advised to dedicate a certain number of sub-pools to the required thinking order. For example, suppose the instructor designs his RSQE to be 10 question exam of 60% lower-order questions and 40% higher-order thinking questions. In that case, 6 sub-pools should be dedicated to lower-order thinking questions, including remembering information, demonstrating understanding, and using the acquired information. At the same time, 4 sub-pools should be dedicated to higher-order thinking questions, including analyzing, discovering, and organizing information, integrating knowledge, and making judgments.
3. The instructor should ensure that each sub-pool has questions of the same difficulty level to the student.
4. Although RSQE effectively interferes with collective memory, the instructor is advised to update the sub-pools following each examination period by including new questions and paraphrasing the exhausted questions to prevent passing examination information to the next batch of students whenever proctoring technologies are limited.

## Conclusions

The RSQE model is considered as a potential solution to mitigate question-sharing between students. Hence, the proper design of RSQE addresses replicated inter-examination and sequential intra-examination questions. By conducting an empirical study through generating 600 RSQEs, this study could address two research questions: (1) How does RSQE design impact the replicated inter-examination question?; (2) How does the RSQE design impact sequential intra-examination questions?. Results revealed that the number of randomly selected questions from the question pool affected their replication at inter-examination level: reduced QPR led to reductions in replicated questions. Moreover, as the number of sub-pools increased, the trend of additional questions with fewer replications increased. Furthermore, RSQE design impacted the sequential intra-examination questions - reduced QPR led to fewer sequential questions, and as the number of sub-pools increased, the trend of sequential questions decreased.

In essence, examiners are advised to design the RSQE in many sub-pools, in equivalence to the number of examination questions and selecting only one question from each sub-pool. They are also advised to consider the QPR to be in the 5–10% range. In addition, examiners are advised to consider sub-pools with questions of equivalent difficulty, update their question pools by including new questions and paraphrase the exhausted questions following every examination period, in order to prevent passing examination information to the next batch of students.

## Data Availability

All data and materials are available.

## Abbreviations

OEP: Online Examination Paper
QPR: Questions/Pool Ratio
QR: Question Repeatability
QS: Question Sequence
RSQE: Randomly Selected Question Examination
SDQ: Sequential Duplicate Questions
SQQ: Sequential Quadratic Questions
STQ: Sequential Triplicate Questions
